# Infectious Complications and Prognostic Factors of Mortality in Patients with Lupus Nephritis Admitted to Intensive Care Units

**DOI:** 10.3390/jcm14217561

**Published:** 2025-10-25

**Authors:** Silvia E. Aldana-Pérez, Diego F. García-Bañol, Adrianny M. Arias-Choles, Gustavo J. Aroca-Martínez, Carlos G. Musso, Alex Dominguez-Vargas, Henry J. González-Torres

**Affiliations:** 1 Centro de Investigaciones en Ciencias de la Vida, Facultad de Ciencias de la Salud, Universidad Simón Bolívar, Barranquilla 080001, Colombia; silvia.aldanap@unisimon.edu.co (S.E.A.-P.); diego.garciab@unisimon.edu.co (D.F.G.-B.); adrianny.arias@unisimon.edu.co (A.M.A.-C.); gustavo.aroca@unisimon.edu.co (G.J.A.-M.); carlos.musso@hospitalitaliano.org.ar (C.G.M.); 2 Data Analysis and Mining Department, D&P Consulting Service SAS, Barranquilla 080001, Colombia; aadominguez17@hotmail.com; 3 Departamento de Nefrología, Clínica de la Costa, Barranquilla 080001, Colombia; 4 Nephrology Department, Hospital Italiano de Buenos Aires, Buenos Aires C1000, Argentina

**Keywords:** lupus nephritis, intensive care units, sepsis, mortality, prognosis, complement C3

## Abstract

**Objective:** To determine infectious complications and explore potential prognostic factors associated with mortality in patients with lupus nephritis (LN) admitted to the intensive care unit (ICU). **Methods:** We conducted a retrospective analytical study of 20 patients with biopsy-proven LN admitted to a tertiary ICU between 2022 and 2023. Clinical, histopathological, microbiological, and paraclinical data were collected. Associations with mortality were explored using Firth’s penalized logistic regression. **Results:** The mean age was 37 ± 14 years; 85% were female. Hypertension (50%) was the most frequent comorbidity. Mean ICU stay was 13 ± 27 days; in-hospital mortality was 15%, and 60% required hospital readmission. Sepsis was the leading reason for ICU admission (55%), predominantly respiratory and gastrointestinal. In the exploratory analysis, respiratory tract infection (OR 1.43; 95% CI: 1.19–9.90; *p* = 0.04), proliferative LN (OR 2.12; 95% CI: 1.32–17.34; *p* = 0.03), and hypocomplementemia (C3) (OR 1.72; 95% CI: 1.25–10.40; *p* = 0.02) showed point estimates suggestive of higher odds of mortality. **Conclusions:** In this cohort of critically ill patients with LN, respiratory tract infection, proliferative histological class, and hypocomplementemia were associated with higher mortality. These findings require validation in larger prospective studies to determine their utility in risk stratification and ICU management.

## 1. Introduction

Lupus nephritis (LN), one of the most severe manifestations of systemic lupus erythematosus (SLE), is characterized by glomerular inflammation that can rapidly progress to irreversible renal damage and kidney failure, requiring aggressive immunosuppressive therapy that increases the risk of severe infections and systemic complications [1,2]. Despite advances in diagnosis, therapeutic management, and supportive care, LN remains associated with high morbidity and mortality, particularly in patients with advanced disease, comorbidities, or acute complications such as sepsis [3,4].

Recent findings in immune-mediated conditions, such as non-infectious uveitis following immune checkpoint inhibitor therapy, reveal that dysregulated immunity can simultaneously drive autoimmune-like tissue injury and increase infection risk [5,6]. This mirrors the dual challenge in LN: underlying autoimmune-driven organ damage compounded by iatrogenic immunosuppression. Exploring these shared pathways may inform better risk stratification and targeted care in critically ill LN patients [5,7]

Patients with SLE and nephritis have an impaired immune system due both to the disease itself and to the immunosuppressive therapies used to control activity [8]. Infections, which may be community-acquired or nosocomial, represent one of the main causes of admission to intensive care units (ICU) in this population [9,10]. A recent review showed that more than 50% of ICU admissions in critically ill SLE patients were due to infections, with substantially higher in-hospital mortality rates among those who developed sepsis [11].

Although this risk is well recognized, the specific factors predisposing LN patients admitted to the ICU to worse outcomes, including clinical, histopathological, paraclinical, and microbiological parameters—remain poorly defined. Studies such as that of Guo et al. in China identified elevated lactate levels, higher APACHE II scores, need for vasopressors, and low platelet counts as independent predictors of mortality in critically ill LN patients [2]. Likewise, recent global cohorts have reported resistant pathogens, hypocomplementemia, and certain proliferative histological classes as potential risk markers for mortality [1,12].

Disease severity at ICU admission, reflected in prognostic scores such as APACHE, SOFA, or other systemic indices, has also been consistently correlated with mortality in critically ill SLE and LN patients [2,4]. Another relevant aspect is the interplay between prior chronic kidney injury, renal function at ICU admission, and the degree of histological involvement, since structural glomerular damage may limit recovery, increase susceptibility to infections, and exacerbate multiorgan dysfunction [2,8].

In Colombia and other Latin American countries, reports on LN in the ICU are scarce, and detailed information integrating microbiological, histopathological, and immunological variables to identify prognostic factors of mortality in critical care settings is lacking. In one Colombian study, ICU mortality reached 33%, with infection as the leading cause of admission (≈55%) and death, followed by disease activity, renal failure, shock at presentation, and low platelet counts as independent predictors [13]. Immunological markers such as anti-dsDNA and complement alterations have been documented, but their relationship with mortality in critical care and severe infections has not been specifically analyzed in this context [14]. These knowledge gaps limit our ability to stratify risk, optimize therapies, prevent infectious complications, and improve outcomes in this vulnerable population.

Therefore, the aim of this study is to determine infectious complications and explore potential prognostic factors associated with mortality in patients with LN admitted to the ICU, including sociodemographic, clinical, paraclinical, histopathological, and microbiological variables. We hypothesize that proliferative histological profiles, complement alterations, severe respiratory infections, and severity scores at ICU admission are independent predictors of mortality. Understanding these factors will support better therapeutic strategies, intensive monitoring, and clinical decision-making in critically ill patients with LN.

## 2. Materials and Methods

### 2.1. Study Design

We conducted a retrospective analytical study aimed at describing infectious complications and potential prognostic factors of mortality in patients with LN admitted to the ICU of a tertiary hospital in Barranquilla, Colombia, between 2022 and 2023.

### 2.2. Population and Inclusion Criteria

The study population consisted of patients older than 18 years with biopsy-proven LN, hospitalized in the ICU during the study period. Patients with incomplete medical records or uncertain LN diagnoses were excluded. A consecutive sampling strategy was used, including all eligible cases within the defined time frame.

### 2.3. Variables and Operational Definitions

Collected variables included sociodemographic data (age, sex), clinical characteristics (comorbidities, disease duration), paraclinical parameters (hematology, renal profile, immunological markers), histopathological findings (ISN/RPS class, activity and chronicity indices), and microbiological data (pathogens isolated, resistance profiles, infection sites). The primary outcome was in-ICU mortality. Secondary outcomes included ICU length of stay and hospital readmission. Sepsis was defined according to the Sepsis-3 criteria.

### 2.4. Data Sources and Collection

Data was retrieved from institutional electronic medical records, complemented by reports from the clinical laboratory, microbiology, and pathology departments. Information was entered into a de-identified database specifically designed for the study, ensuring patient confidentiality.

### 2.5. Statistical Analysis

Normality of continuous variables was assessed using the Shapiro–Wilk test. Continuous variables were expressed as means with standard deviations or medians with ranges, as appropriate. Categorical variables were summarized as absolute and relative frequencies. Comparisons between groups (sex, proliferative vs. non-proliferative histological class) were performed using Student’s *t*-test for independent samples or its non-parametric equivalent. Changes in paraclinical parameters between ICU admission and discharge were evaluated with paired Student’s *t*-test. Categorical variables were compared using chi-square or Fisher’s exact test when expected frequencies were <5. A penalized multivariate logistic regression (Firth’s method) was used to explore potential predictors of in-hospital mortality, a set of clinically plausible predictors were selected *a priori* based on pathophysiological relevance and was included in the model. Results are presented as odds ratios (ORs) with 95% profile penalized likelihood confidence intervals. A *p* value < 0.05 was considered statistically significant. All analyses were performed with R-CRAN software, version 4.3.3 [15].

## 3. Results

### 3.1. General Characteristics

A total of 20 patients with a diagnosis of lupus nephritis (LN) admitted to an intensive care unit (ICU) were included in this study. The mean age was 37 ± 14 years, and most patients were female (85%) with an average disease duration of 5.3 ± 5.2 years (Table 1).

**Figure 1:**
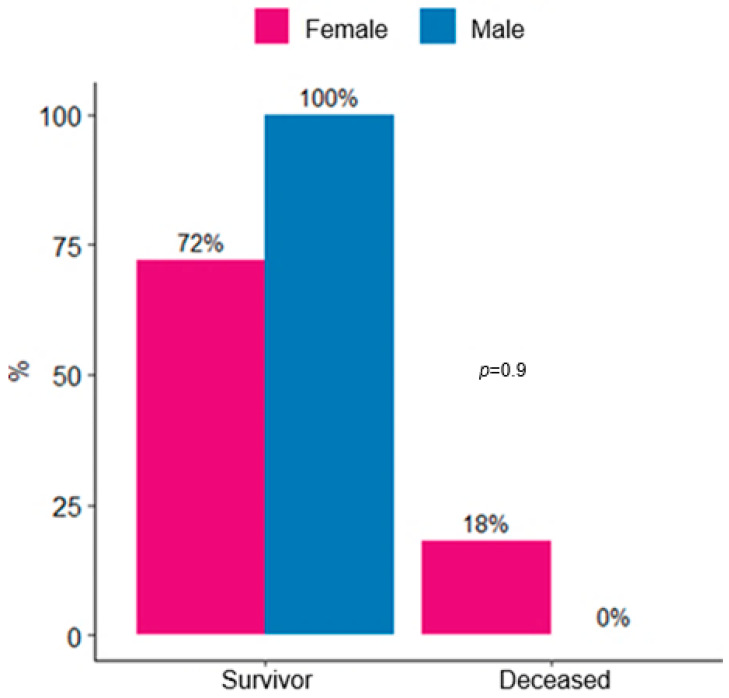
Distribution of survivors and deaths in lupus nephritis patients admitted to ICU by sex.

The most frequent comorbidity was hypertension (HTN), present in 50% of patients, followed by heart failure (HF) (20%) and hypothyroidism (15%). The mean ICU length of stay was 13 ± 27 days. In this cohort, 12 patients (60%) required hospital readmission and 3 (15%) died. No significant differences were observed in mortality according to sex (female 18% vs. male 0%, *p* = 0.9) (Figure 1).

### 3.2. Admission Diagnosis

Table 2 presents the most frequent admission diagnoses. Sepsis was predominant, affecting 11 patients (55%). Among septic cases, three were gastrointestinal (15%), three respiratory (15%), two urinary tract (10%), one central nervous system (5%), one mixed (central venous catheter and pneumonia) (5%), and one renal (5%). In addition, 2 patients (10%) were admitted for decompensated heart failure and 2 (10%) for hypertensive emergencies.

### 3.3. Histopathology and Treatment

Table 3 compares histopathological profiles and treatments by sex. Mean age was similar in both groups: 37 ± 14 years in females and 37 ± 16 years in males (*p* > 0.9). Proliferative classes were the most frequent overall (55%). Class IV was the predominant histological subtype, observed in 59% of females and 67% of males (*p* > 0.9). Class II was present in 18% of females, while Class V was identified in 23% of females and in one male patient.

Regarding immunosuppressive therapy, the use of cyclophosphamide (CYC) and mycophenolate mofetil (MMF) was higher in males (33% each) than in females (18% each), though this difference was not statistically significant (*p* = 0.51 and *p* = 0.53, respectively). Hydroxychloroquine (HCQ) was more frequently used in females (65% vs. 33% in males; *p* = 0.54), while azathioprine (AZA) was prescribed only to females (12%; *p* > 0.9). All patients in our cohort received corticosteroids during ICU admission, consistent with standard care for severe LN. The typical regimen included intravenous methylprednisolone pulses (250–500 mg/day for 3 days) followed by oral prednisone (0.8–1 mg/kg/day) [16].

**Figure 2:**
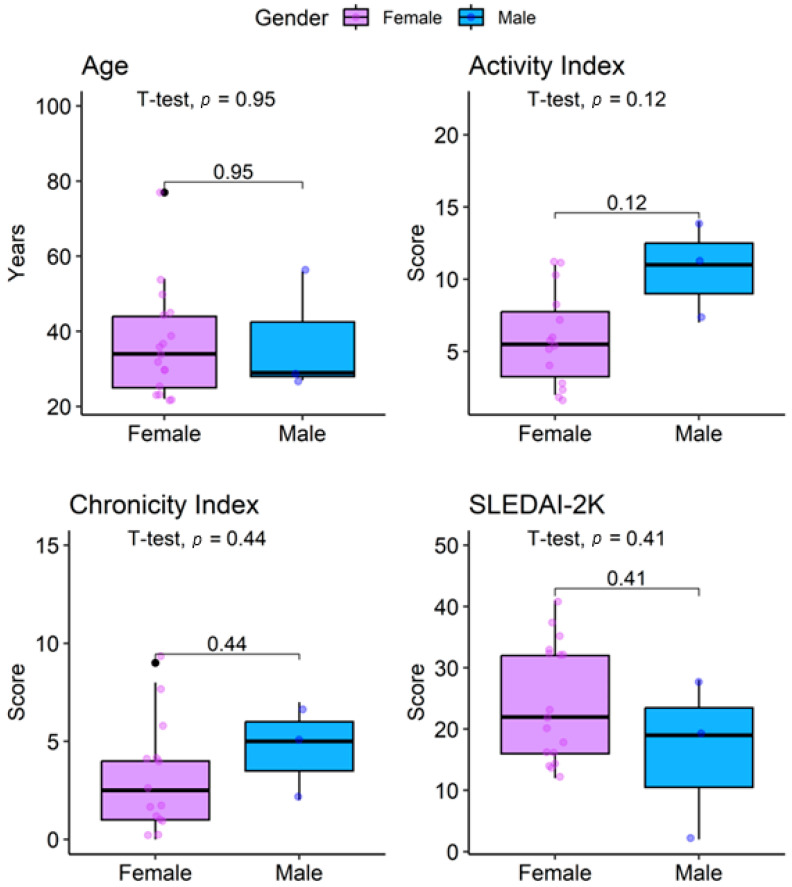
Distribution of age, activity index, chronicity index, and SLEDAI-2K in lupus nephritis patients by sex.

The activity index was higher in males (10.5 ± 4.9) compared with females (5.8 ± 3.2), although not statistically significant (*p* = 0.12). Similarly, the chronicity index was slightly higher in males (4.5 ± 3.5 vs. 3.2 ± 2.8, *p* = 0.44). Conversely, the SLEDAI-2K score was higher in females (24 ± 10 vs. 16 ± 13, *p* = 0.4) (Figure 2).

### 3.4. Microbiological Profile

**Figure 3:**
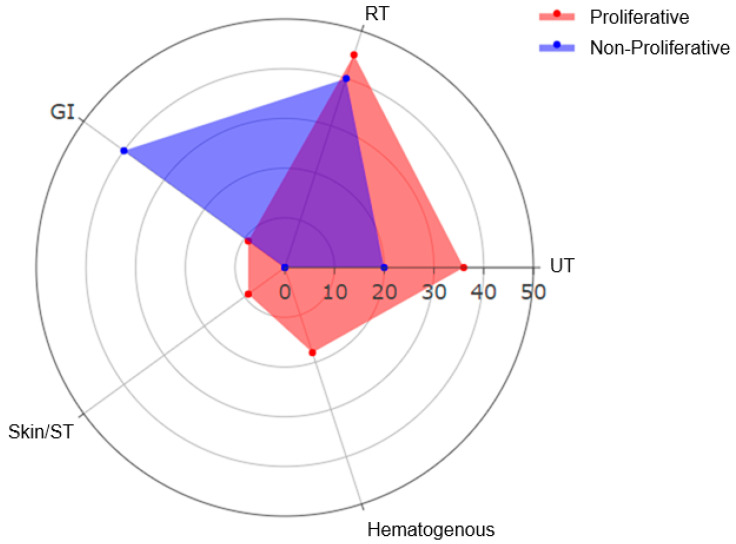
Distribution of infection sites in lupus nephritis ICU patients according to histopathology (RT: respiratory tract, GI: gastrointestinal; UT: Urinary Tract; Skin/ST: Skin/Soft Tissue).

Microbiological profiles were compared between proliferative and non-proliferative classes (Table 4). Urinary tract infections were more frequent in proliferative LN (36% vs. 20%), though not significant (*p* > 0.92). Respiratory tract infections were also more frequent in the proliferative group (45% vs. 40%, *p* > 0.92). Hematogenous and skin/soft tissue infections were observed exclusively in the proliferative group (Figure 3).

In terms of pathogens, non-proliferative cases showed *Escherichia coli* (33%), *Mycobacterium tuberculosis* (33%), and *Staphylococcus epidermidis* (33%). In the proliferative group, *Klebsiella pneumoniae* (25%) and *E. coli* (25%) predominated. Other pathogens, including *Cryptococcus gattii*, *Enterobacter hormaechei*, and *Staphylococcus aureus*, were found only in proliferative LN, though differences were not statistically significant (*p* = 0.72).

Complicated infections were more common in the proliferative group (71% vs. 50%, *p* = 0.62). All infections in non-proliferative cases were antibiotic-sensitive, whereas extended-spectrum beta-lactamase (ESBL)-producing organisms and methicillin-resistant Staphylococcus aureus (MRSA) were detected in 13% of proliferative cases (*p* > 0.92).

### 3.5. Hematological Profile

Table 5 shows hematological parameters at ICU admission and discharge. No statistically significant differences were observed in any parameter (all *p* > 0.05). Red blood cells decreased slightly from 3.16 ± 1.00 × 10^6^/mm^3^ at admission to 3.02 ± 0.54 × 10^6^/mm^3^ at discharge (*p* = 0.47). Hemoglobin (Hb) remained stable (8.23 ± 2.04 g/dL at admission vs. 8.24 ± 1.61 g/dL at discharge, *p* = 0.98).

**Figure 4:**
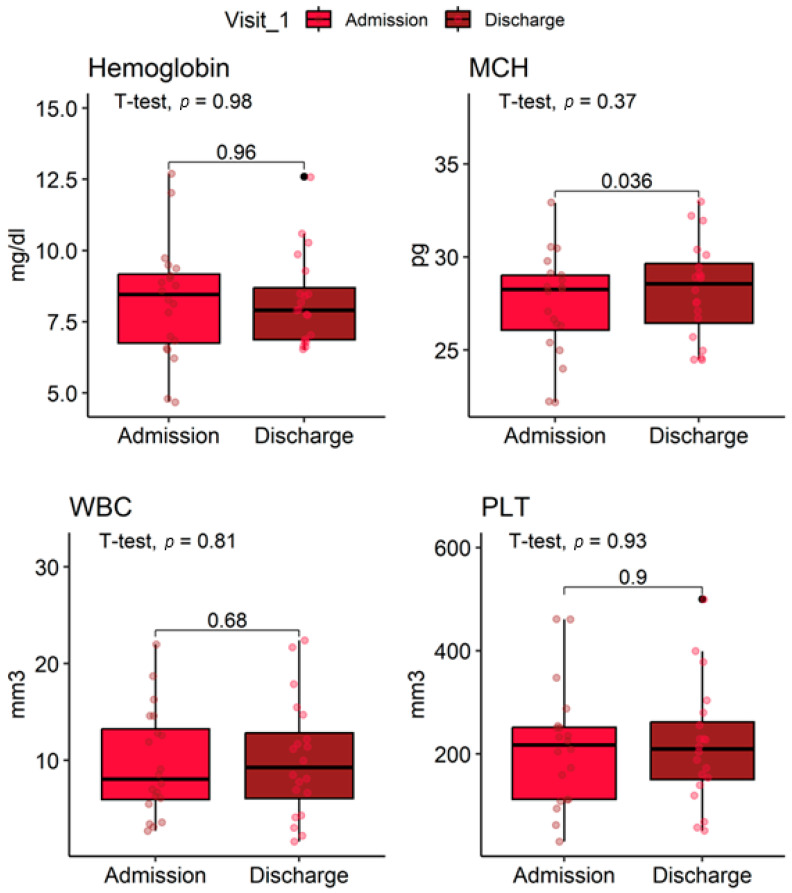
Distribution of hematological parameters at ICU admission and discharge in lupus nephritis patients.

Mean corpuscular volume (MCV) remained stable (83 ± 7 fL vs. 82.9 ± 5.5 fL, *p* = 0.82). Mean corpuscular hemoglobin (MCH) increased slightly (27.46 ± 2.75 pg vs. 28.23 ± 2.59 pg, *p* = 0.37). Mean corpuscular hemoglobin concentration (MCHC) was unchanged (33.03 ± 1.58 g/dL vs. 33.09 ± 1.68 g/dL, *p* = 0.87). White blood cell count was stable (9.6 ± 5.5 ×10^3^/mm^3^ vs. 9.6 ± 6.4 ×10^3^/mm^3^, *p* = 0.98). Platelet counts remained similar (213 ± 116 ×10^3^/mm^3^ vs. 216 ± 115 ×10^3^/mm^3^, *p* = 0.90) (Figure 4).

### 3.6. Renal Function and Immunological Profile

Non-proliferative LN patients had a mean eGFR of 49 ± 31 mL/min/m^2^, compared with 43 ± 35 mL/min/m^2^ in proliferative cases, with no significant difference (*p* = 0.53) (Table 6). In contrast, patients with proliferative LN exhibited significantly higher frequencies of positive anti-dsDNA (58% vs. 25%), anti-C1q (50% vs. 13%), and anti-Sm (58% vs. 13%) antibodies compared to those with non-proliferative LN (all *p* < 0.05).

**Figure 5:**
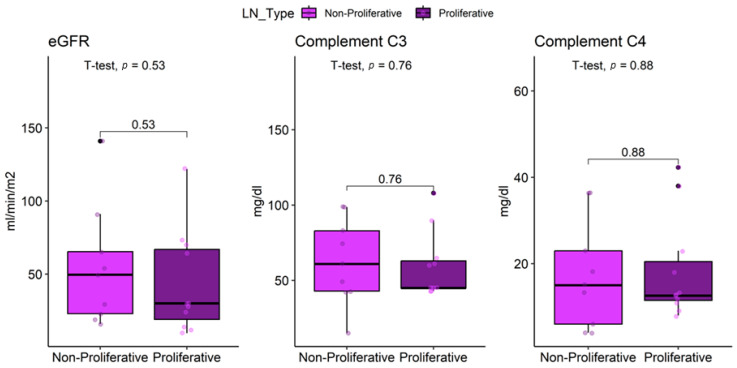
Comparison of eGFR, complement C3 and C4 in lupus nephritis patients by histological profile.

C3 levels were 65 ± 23 mg/dL in proliferative LN and 51 ± 35 mg/dL in non-proliferative LN (*p* = 0.52). Hypocomplementemia C3 was equally prevalent in both groups (75%, *p* > 0.93). C4 levels were 18 ± 14 mg/dL in proliferative LN vs. 15 ± 15 mg/dL in non-proliferative LN (*p* = 0.88). Hypocomplementemia C4 was present in 50% of non-proliferative and 38% of proliferative cases (*p* > 0.93) (Figure 5).

### 3.7. Factors Associated with Mortality (Exploratory Analysis)

Mortality during ICU admission occurred in 3 of 20 patients (15%) with LN. Given the limited number of events, an exploratory penalized logistic regression (Firth’s method) was performed to assess potential associations with baseline demographic, clinical, and serological factors. In this hypothesis-generating analysis, respiratory tract infection (OR 1.43; 95% CI 1.19–9.9; *p* = 0.04), proliferative lupus nephritis (OR 2.12; 95% CI 1.32–17.34; *p* = 0.03), and hypocomplementemia (C3 < 90 mg/dL; OR 1.72; 95% CI 1.25–10.4; *p* = 0.02) suggested increased odds of mortality (Table 7).

## 4. Discussion

In this retrospective analytical study of 20 patients with LN admitted to an ICU, we found a high burden of infectious complications and a mortality rate of 15%. These findings are consistent with previous reports describing infections as the leading cause of ICU admission and adverse outcomes in this population [8,9]. The majority of our patients were young women (mean age 37 years), which is in line with the classic epidemiology of SLE and its renal manifestations [17,18].

More than half of ICU admissions were due to sepsis (55%), primarily of respiratory and gastrointestinal origin. This is consistent with international cohorts, where sepsis accounts for 40–60% of admissions in critically ill SLE patients [3,10]. In a multicenter study from China, respiratory sepsis was identified as an independent predictor of mortality in LN, similar to our adjusted model [2]. Specifically, respiratory infections in our series yielded a 1.4-fold higher odds of death, highlighting the vulnerability of this population to severe pneumonia, likely exacerbated by immunosuppressive therapy, mechanical ventilation, and invasive devices [11,19].

Proliferative class IV LN was the most common histological form, consistent with Latin American and global reports, where proliferative classes represent the majority of severe LN cases requiring hospitalization [1,13]. In our study, proliferative LN was associated with more frequent and severe infections, supporting the concept of a “double burden” of risk: severe inflammatory activity coupled with intensive immunosuppression increases susceptibility to infections [20,21]. Proliferative LN showed a point estimate suggesting a 2.1-fold higher odds of death consistent with findings by Guo et al. in critically ill SLE patients [2].

Hematological profiles at ICU admission and discharge revealed no statistically significant changes across any measured parameter, including red blood cell indices, white blood cell subtypes, and platelet counts. While this may initially appear unremarkable, it carries potential clinical relevance in the context of severe LN. In critically ill patients, one might anticipate progressive anemia, leukocytosis, or thrombocytopenia due to ongoing inflammation, sepsis, or immunosuppressive effects, yet none were observed here [22,23].

The relative stability of hemoglobin, hematocrit, and mean corpuscular indices suggests that anemia in this cohort was chronic rather than acute-onset, possibly reflecting baseline SLE-related bone marrow suppression or renal insufficiency [24]. Similarly, the lack of neutrophilic shift or lymphopenia despite high rates of infection implies preserved innate immune responsiveness or potentially masked by concurrent corticosteroid use [25]. Platelet counts remained stable, which contrasts with prior reports linking low platelets to mortality in SLE [6], suggesting that in this ICU population, platelet levels alone may not be a dynamic prognostic marker over short-term hospitalization.

Our findings align with emerging evidence that distinct autoantibody profiles are associated with specific histological subtypes of LN. Consistent with prior studies [26,27], patients with proliferative LN in our cohort exhibited significantly higher prevalences of anti-dsDNA, anti-C1q, and anti-Sm antibodies. These associations are biologically plausible: anti-dsDNA and anti-C1q antibodies are known to form immune complexes in the glomeruli, triggering inflammation and complement activation, which underlie the proliferative lesions characteristic of Class III/IV LN [26,28]. In contrast, autoantibodies such as anti-RNP, anti-Ro/SSA, and anti-La/SSB showed no significant differences between histological subtypes consistent with reports that these are more frequently associated with milder or extrarenal manifestations, including membranous nephritis (Class V), where anti-RNP70 positivity has been specifically linked [26].

Hypocomplementemia C3 yielded a 1.7-fold higher odds of death, aligning with prior evidence linking low complement levels not only to disease activity but also to increased risk of severe infections and fatal outcomes [29,30,31,32]. Recent studies have emphasized that low C3 levels should be considered a prognostic biomarker in both renal and systemic disease activity, reinforcing its role in risk stratification for critically ill LN patients [33].

The observed mortality (15%) in this study was lower than reported in other Latin American cohorts: a single-center study from Argentina found 55% mortality among critically ill SLE patients, primarily driven by infectious complications [34]; in Brazil, Ranzani et al. reported 20–33% ICU mortality across systemic rheumatic diseases, with SLE patients showing 20% mortality [35]; and in Mexico, Ñamendys-Silva et al. documented 32.7% mortality in 104 SLE patients admitted to ICU, with infection as the leading cause of admission and high APACHE II score and vasopressor use as key predictors [36]. These differences may reflect variations in patient selection, timing of ICU admission, or access to immunosuppressive protocols. For instance, all patients in our cohort received corticosteroid therapy during ICU admission, consistent with standard management of severe LN [16]. Notably, our rate aligns with improving trends in Mexico, suggesting advances in critical care may be reducing mortality regionally [36]. Nevertheless, the high hospital readmission rate (60%) in our cohort underscores the fragility of this population and reinforces the urgent need for close post-discharge monitoring and preventive strategies [37].

In Colombia, previous studies have reported ICU mortality close to 30% among SLE patients, with infections as the main cause of admission and death [38]. The Medellín cohort identified predictors such as thrombocytopenia, shock at admission, and acute kidney injury [38], partially overlapping with our findings. However, few local studies have integrated microbiological, histopathological, and immunological parameters in a single model, which constitutes a novel contribution of our work. The identification of respiratory sepsis, proliferative histology, and hypocomplementemia C3 as independent predictors of mortality highlights the need for proactive infection surveillance, early pathogen detection, and rational antibiotic use in critically ill LN patients [21,39,40]. Integrating these factors into risk stratification could support personalized management strategies and guide therapeutic decisions in high-risk cases.

This study has limitations, including its retrospective design, which may introduce selection and information bias, as well as its single center setting and small sample size, which restricts generalizability to broader populations of critically ill LN patients. Although consecutive sampling enhances internal validity, it may not capture the full heterogeneity of disease severity or comorbidities seen in multicenter cohorts. Additionally, the lack of standardized severity-of-illness scoring (e.g., APACHE II or SOFA) and incomplete documentation of renal biopsy immunofluorescence findings (e.g., C3, IgG deposits) limits direct comparison with international studies. Nevertheless, a major strength lies in the integration of clinical, microbiological, histopathological, and immunological variables into a single prognostic analysis, offering a more comprehensive perspective on outcomes in this vulnerable population

Our results suggest that respiratory sepsis, proliferative histology, and hypocomplementemia C3 should be considered key risk indicators in LN patients admitted to the ICU. Larger multicenter studies are needed to validate these findings and to develop predictive models that can inform clinical decision-making and improve outcomes in this vulnerable population. The identification of these robust clinical and immunological predictors provides a foundation for future research incorporating advanced analytical methods. In particular, machine learning approaches such as bagging, histogram-based gradient boosting, and automated feature selection have shown promise in predicting complex health outcomes in autoimmune and critical care settings [41,42]. Application of these techniques in larger LN cohorts could facilitate the development of dynamic, individualized risk prediction tools to guide precision management.

## 5. Conclusions

In this cohort of patients with LN admitted to ICU, sepsis was the leading cause of admission and was associated with a high burden of complications, with a mortality rate of 15% and 60% hospital readmissions. Respiratory tract infection, proliferative histological class, and hypocomplementemia C3 showed point estimates suggestive of increased mortality risk. These findings should be interpreted as hypothesis-generating rather than confirmatory. Nevertheless, they are biologically plausible and consistent with the recognized “double burden” of severe LN, where intense inflammatory activity and immunosuppressive therapy increase susceptibility to life-threatening infections.

While these signals do not support definitive changes in practice, they underscore the potential value of enhanced clinical surveillance, careful immunosuppression titration, and early infection prevention strategies in critically ill LN patients. From a clinical and public health perspective, integrating microbiological monitoring and antimicrobial stewardship into ICU care pathways for lupus may be warranted, though their impact remains to be tested. Future multicenter, prospective studies with sufficient statistical power are essential to validate these associations and to develop reliable predictive models for risk stratification and personalized management in this vulnerable population.

## Figures and Tables

**Table 1:** General characteristics of patients with lupus nephritis.

Characteristic	Value ^1^
Age (years)	37 ± 14
Sex	
Female	17 (85%)
Male	3 (15%)
Disease duration, years	5.3 ± 5.2
Comorbidities	
Hypertension	10 (50%)
Hypothyroidism	3 (15%)
Heart failure	4 (20%)
ICU stay, days	13 ± 27
Outcomes	
Mortality	3 (15%)
Hospital readmission	12 (60%)

ICU, intensive care unit; ^1^ Mean ± SD; n (%).

**Table 2:** Admission diagnosis in lupus nephritis patients admitted to ICU.

Diagnosis	Value ^1^
Sepsis	11 (55)
GI	3 (15)
RT	3 (15)
UT	2 (10)
CNS	1 (5)
Mixed	1 (5)
Renal	1 (5)
DHF	2 (10)
Hypertensive emergency	2 (10)

GI, gastrointestinal; RT, respiratory tract; UT, urinary tract; CNS, central nervous system; DHF, decompensated heart failure; ^1^ n (%).

**Table 3:** Histopathological profile and treatment in lupus nephritis patients.

Parameter	Female (n = 17) ^1^	Male (n = 3) ^1^	*p*-Value
Age, years	37 ± 14	37 ± 16	>0.93 ^2^
LN class			>0.94 ^3^
Class II	3 (18)	0 (0)	
Class IV	10 (59)	2 (67)	
Class V	4 (23)	1 (33)	
Activity index	5.8 ± 3.2	10.5 ± 4.9	0.12 ^2^
Chronicity index	3.2 ± 2.8	4.5 ± 3.5	0.44 ^2^
SLEDAI-2K, score	24 ± 10	16 ± 13	0.40 ^2^
Immunosuppressive therapy			0.9 ^3^
CYC	3 (18)	1 (33)	0.51 ^3^
MMF	3 (18)	1 (33)	0.53 ^3^
HCQ	11 (65)	1 (33)	0.54 ^3^
AZA	2 (12)	0 (0)	>0.92 ^3^

LN, lupus nephritis; CYC, cyclophosphamide; MMF, mycophenolate mofetil; HCQ, hydroxychloroquine; AZA, azathioprine; CT: corticosteroids; SLEDAI-2K, Systemic Lupus Erythematosus Disease Activity Index 2000. ^1^ Mean ± SD; n (%); ^2^ Welch Two Sample *t*-test; ^3^ Fisher’s exact test.

**Table 4:** Microbiological profile according to histological class in lupus nephritis patients.

Parameter	Non-Proliferative (N = 8) ^1^	Proliferative (N = 12) ^1^	*p*-Value
Infection site			
UT	1 (20)	4 (36)	>0.92 ^2^
RT	2 (40)	5 (45)	>0.93 ^2^
GI	2 (40)	1 (9.1)	0.21 ^2^
Skin/soft tissue	0 (0)	1 (9.1)	>0.96 ^2^
Hematogenous	0 (0)	2 (18)	>0.94 ^2^
Infectious agent			0.71 ^2^
*Cryptococcus Gatti*	0 (0)	1 (13)	
*E. Coli*	1 (33)	2 (25)	
*Enterobacter hormaechei*	0 (0)	1 (13)	
*Klebsiella pneumoniae*	0 (0)	2 (25)	
*Mycobacterium tuberculosis*	1 (33)	1 (13)	
*Staphylococcus aureus*	0 (0)	1 (1%)	
*Staphylococcus epidermidis*	1 (33)	0 (0)	
Severity			0.64 ^2^
Complicated	2 (50)	5 (71)	
Severe	2 (50)	2 (29)	
Resistance profile			>0.94 ^2^
ESBL	0 (0)	1 (13)	
MRSA	0 (0)	1 (13)	
Susceptible	3 (100)	6 (75)	

GI: gastrointestinal; RT: respiratory tract; UT: urinary tract; MRSA: methicillin-resistant *Staphylococcus aureus*; ESBL: extended-spectrum beta-lactamase; ^1^ n (%); ^2^ Fisher’s exact test.

**Table 5:** Comparison of hematological parameters at ICU admission and discharge in lupus nephritis patients.

Parameter	Admission (N = 20) ^1^	Discharge (N = 20) ^1^	*p*-Value ^2^
RBC, ×10^6^/mm^3^	3.16 ± 1.00	3.02 ± 0.54	0.47
Hemoglobin, g/dL	8.23 ± 2.04	8.24 ± 1.61	0.98
Hematocrit, %	24.0 ± 5.3	24.6 ± 4.6	0.63
MCV, fL	83 ± 7	82.9 ± 5.5	0.82
MCH, pg	27.46 ± 2.75	28.23 ± 2.59	0.37
MCHC, g/dL	33.03 ± 1.58	33.09 ± 1.68	0.87
Leukocytes, ×10^3^/mm^3^	9.6 ± 5.5	9.6 ± 6.4	0.98
Neutrophils, %	47 ± 25	50 ± 30	0.67
Monocytes, ×10^3^/mm^3^	431 ± 389	411 ± 335	0.85
Eosinophils, ×10^3^/mm^3^	262 ± 182	193 ± 236	0.53
Basophils, ×10^3^/mm^3^	34 ± 24	46 ± 34	0.86
Platelets, ×10^3^/mm^3^	213 ± 116	216 ± 115	0.9
MPV, fL	10.2 ± 1.5	9.6 ± 1.4	0.12

RBC, red blood cells; MCV, mean corpuscular volume; MCH, mean corpuscular hemoglobin; MCHC, mean corpuscular hemoglobin concentration; MPV, mean platelet volume; ^1^ Mean ± SD; ^2^ Welch Two Sample paired *t*-test.

**Table 6:** Renal function and immunological parameters in lupus nephritis patients by histological profile.

Parameter	Non-Proliferative (N = 8) ^1^	Proliferative (N = 12) ^1^	*p*-Value
Renal Function			
eGFR (ml/min/m^2^)	49 ± 31	43 ± 35	0.53 ^2^
Aab positivity			
dsDNA	2 (25)	7 (58)	0.04 ^3^
C1q	1 (13)	6 (50)	0.03 ^3^
Sm	1 (13)	7 (58)	0.03 ^3^
RNP	3 (38)	6 (50)	0.24 ^3^
Ro/SSA	4 (50)	5 (42)	0.41 ^3^
La/SSB	3 (38)	3 (25)	0.62 ^3^
Complement			
C3, mg/dL	51 ± 35	65 ± 23	0.5 ^2^
Hypocomplementemia C3	3 (75)	6 (75)	>0.9 ^3^
C4, mg/dL	15 ± 15	18 ± 14	0.88 ^2^
Hypocomplementemia C4	2 (50)	3 (38)	>0.9 ^3^

eGFR: Estimated Glomerular Filtration Rate; Aabs: autoantibodies; C3: Complement C3; C4: Complement C4; ^1^ Mean ± SD; n (%); ^2^ Welch Two Sample *t*-test; ^3^ Fisher’s exact test.

**Table 7:** Exploratory factors linked to mortality in ICU lupus nephritis.

Parameter	OR ^1^	95% CI ^1^	*p*-Value
Age > 35 years	9.9	0.6–15.5	0.6
Male sex	0.24	0.1–9.29	0.9
Respiratory Tract Infection	1.43	1.19–9.9	0.04
Proliferative Lupus Nephritis	2.12	1.32–17.34	0.03
SLEDAI-2K > 30	3.6	0.31–23.5	0.7
Hypocomplementemia C3	1.72	1.25–10.4	0.02

SLEDAI-2K: Systemic Lupus Erythematosus Disease Activity Index 2000; ICU: intensive care unit. ^1^ OR = Odds Ratio (Firth penalized logistic regression); CI = confidence interval.

## Data Availability

All data supporting the findings of this study are included in this article. Anonymized datasets underlying the results may also be provided by the authors upon reasonable written request with a justified purpose of use.
